# Functional Correlates of Self-Reported Energy in the Health, Aging, and Body Composition Study

**DOI:** 10.1093/geroni/igaa057.558

**Published:** 2020-12-16

**Authors:** Rebecca Ehrenkranz, Andrea Rosso, Briana Sprague, Qu Tian, Eleanor Simonsick, Nancy Glynn, Caterina Rosano, Theresa Gmelin

**Affiliations:** 1 University of Pittsburgh, Pittsburgh, Pennsylvania, United States; 2 School of Public Health, University of Pittsburgh, Pittsburgh, Pennsylvania, United States; 3 National Institute on Aging, Bethesda, Maryland, United States

## Abstract

While fatigue in older age is well studied, the clinical relevance of maintaining higher energy late in life is less understood. We explored associations of self-reported energy with cognitive performance, depressive symptoms, and physical function in the Health, Aging and Body Composition study (n=2,529, mean age =75.9, 63.5% white, 44.9% men). Self-reported energy over the past month was recorded from 0-10 (least to most energy) and dichotomized at the median (≥7=high energy). Cognitive performance was measured using Modified Mini-Mental State Examination and Digit Symbol Substitution Test. Depressive symptoms were measured using the Center for Epidemiologic Studies Depression scale. Physical function was assessed via fitness (timed 400-meter walk), self-reported physical activity, and usual and rapid gait speed. Variables bivariately associated with energy entered a logistic regression model with higher energy as the outcome, adjusted for demographics, chronic conditions, strength, and body mass index (BMI). Overall, 58% of the sample reported high energy, and self-reported energy was greater for males and those without chronic conditions (p<0.05). Lower odds of higher self-reported energy were found for participants with more depressive symptoms (aOR 95% CI= 0.55 [0.50, 0.62]) and longer time to walk 400m (aOR = 0.79 [0.70, 0.89]). Increased odds of higher self-reported energy were found for participants with faster usual and rapid gait speeds (aOR = 1.3 [1.2, 1.5]; aOR = 1.2 [1.1 – 1.4], respectively). Associations with cognitive performance were not significant. Higher self-reported energy reflects fewer depressive symptoms and greater physical function independent of demographics, chronic conditions, strength, and BMI.

